# *Sirh7/Ldoc1* knockout mice exhibit placental P4 overproduction and delayed parturition

**DOI:** 10.1242/dev.114520

**Published:** 2014-12-15

**Authors:** Mie Naruse, Ryuichi Ono, Masahito Irie, Kenji Nakamura, Tamio Furuse, Toshiaki Hino, Kanako Oda, Misho Kashimura, Ikuko Yamada, Shigeharu Wakana, Minesuke Yokoyama, Fumitoshi Ishino, Tomoko Kaneko-Ishino

**Affiliations:** 1 Department of Epigenetics, Medical Research Institute, Tokyo Medical and Dental University, 1-5-45 Yushima, Bunkyo-ku, Tokyo 113-8510, Japan; 2 School of Health Sciences, Tokai University, Bohseidai, Isehara, Kanagawa 259-1193, Japan; 3 Mitsubishi Kagaku Institute of Life Sciences, 11 Minamiooya, Machida, Tokyo 194-8511, Japan; 4 Faculty of Medicine, Tokai University, Bohseidai, Isehara, Kanagawa 259-1193, Japan; 5 Technology and Development Team for Mouse Phenotype Analysis, The Japan Mouse Clinic, RIKEN BRC, 3-1-1 Koyadai, Tsukuba, Ibaraki 305-0074, Japan; 6 Department of Biological Sciences, Asahikawa Medical University, 2-1-1-1 Midorigaoka-higashi, Asahikawa 078-8510, Japan; 7 Brain Research Institute, Niigata University, 1-757 Asahimachi-dori, Niigata 951-8585, Japan; 8 Global Center of Excellence Program for International Research Center for Molecular Science in Tooth and Bone Diseases, Tokyo Medical and Dental University, 1-5-45 Yushima, Bunkyo-ku, Tokyo 113-8510, Japan

**Keywords:** Evolution, Gestation, Parturition, Placenta, Progesterone, Retrotransposon

## Abstract

*Sirh7/Ldoc1* [sushi-ichi retrotransposon homolog 7/leucine zipper, downregulated in cancer 1, also called mammalian retrotransposon-derived 7 (*Mart7*)] is one of the newly acquired genes from LTR retrotransposons in eutherian mammals. Interestingly, *Sirh7/Ldoc1* knockout (KO) mice exhibited abnormal placental cell differentiation/maturation, leading to an overproduction of placental progesterone (P4) and placental lactogen 1 (PL1) from trophoblast giant cells (TGCs). The placenta is an organ that is essential for mammalian viviparity and plays a major endocrinological role during pregnancy in addition to providing nutrients and oxygen to the fetus. P4 is an essential hormone in the preparation and maintenance of pregnancy and the determination of the timing of parturition in mammals; however, the biological significance of placental P4 in rodents is not properly recognized. Here, we demonstrate that mouse placentas do produce P4 in mid-gestation, coincident with a temporal reduction in ovarian P4, suggesting that it plays a role in the protection of the conceptuses specifically in this period. Pregnant *Sirh7/Ldoc1* knockout females also displayed delayed parturition associated with a low pup weaning rate. All these results suggest that *Sirh7/Ldoc1* has undergone positive selection during eutherian evolution as a eutherian-specific acquired gene because it impacts reproductive fitness via the regulation of placental endocrine function.

## INTRODUCTION

Over 30 genes are derived from long terminal repeat (LTR) retrotransposons in two viviparous mammalian groups: the eutherians and marsupials (Miller et al., 1999; Brandt et al., 2005; Schuller et al., 2005; Youngson et al., 2005; Campillos et al., 2006; Volff, 2006; Ono et al., 2011; Kaneko-Ishino and Ishino, 2012; Iwasaki et al., 2013). Among them, we previously demonstrated that *Peg10* and *Peg11* (*Rtl1* – Mouse Genome Informatics) of the SIRH family play essential roles in mouse development via the formation and maintenance of the placenta, respectively (Ono et al., 2006; Kagami et al., 2008; Sekita et al., 2008). This led to the hypothesis that the SIRH family of genes has powerfully contributed to the evolution of the mammalian viviparous system as newly acquired genes that are active in multiple aspects of placental function, including the maintenance of pregnancy and parturition control as well as maternal behavior and lactation. As *Sirh7/Ldoc1*, another SIRH gene specific to the eutherians, is also highly expressed in the early stages of placental development, we addressed its biological role in mouse development and reproduction by analyzing the phenotype of *Sirh7*/*Ldoc1* knockout (KO).

Placental shape and structure, as well as its endocrine regulatory pathways, exhibit a high degree of diversity across mammalian species (Rossant and Cross, 2001; Malassine et al., 2003; Freyer and Renfree, 2009). In humans, progesterone (P4) is first produced in the ovary and then in the placenta after 8 weeks of gestation, whereas in rodents the ovary was thought to be the major P4 productive organ throughout the course of pregnancy (Malassine et al., 2003). In mice, the serum P4 concentration increases just after mating and reaches a plateau by gestational day 2.5-3.5 [d2.5-3.5, also known as embryonic (E) day], exhibiting an abrupt fall during d9.5-10.5, presumably owing to a shift from the corpus luteum of pseudopregnancy to pregnancy (Murr et al., 1974; Virgo and Bellward, 1974; Malassine et al., 2003). It maintains a higher level from d12.5 to the end of pregnancy and its rapid decrease from d16.5-18.5 is known to be a signal of parturition (Murr et al., 1974; Virgo and Bellward, 1974). However, it still remains unclear why the serum P4 level is specifically downregulated during the period d9.5-10.5 and how embryos overcome the resulting low serum P4 level, as P4 is essential for the maintenance of pregnancy.

Previous reports have demonstrated the expression of a mouse P4 synthesis gene, 3-β-hydroxysteroid dehydrogenase/Δ-5-4 isomerase (*Hsd3b*), in the decidua just after implantation (on d6.5-7.5) and then in placental giant trophoblast cells (TGCs), which are non-dividing polyploid cells formed by endoreduplication, as well as the direct detection of P4 in the culture medium of d9.5 and 10.5 placentas (Arensburg et al., 1999; Peng et al., 2002). However, the biological roles of placental P4 as well as its very presence in rodents have long been under debate due to the lack of a reliable method for assaying P4 in organs directly, except in serum. In the course of an analysis of *Sirh7/Ldoc1* KO mice, we found that pregnant females exhibited delayed parturition due to the high serum P4 concentration on d18.5. Then, we improved the method of determining P4 in tissues and organs directly using mass spectrography and compared the patterns in the ovary and placenta with normal controls. Importantly, we confirmed that the placenta produces P4 specifically during d9.5-12.5, suggesting an essential role of placental P4 in rodent development. Moreover, overproduction of placental P4 and placental lactogen 1 (PL1) was confirmed in *Sirh7/Ldoc1* KO mice, suggesting that these placental hormones play some role on the regulation of timing of parturition. These results support the idea that *Sirh7/Ldoc1* is another essential acquired gene in the establishment of the current viviparous system in the eutherians because it can regulate placental endocrine functions via controlling differentiation and maturation of a wide variety of placental cells.

## RESULTS

### *Sirh7/Ldoc1* KO mice exhibited abnormal placental cell differentiation/maturation

**Fig. 1.**
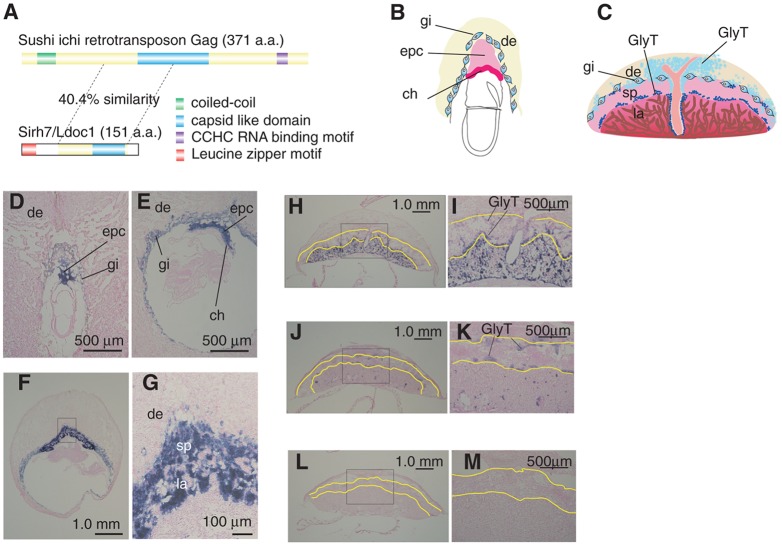
**Placental expression of retrotransposon-derived *Sirh7/Ldoc1*.** (A) Structure of the Sirh7/Ldoc1 protein: 28.3% identity and 40.4% similarity to the sushi-ichi Gag protein were observed in the entire Sirh7/Ldoc1 protein except for the N terminus. The leucine zipper motif (red) in the N terminus of Sirh7/Ldoc1 is a type of coiled-coil structural motif (green) that appears in the N terminus of the Gag protein. The blue and purple colors indicate typical sushi-ichi retrotransposon Gag motifs (a capsid like domain and CCHC RNA-binding motif, respectively), while yellow indicates the parts lacking any characteristic motifs. (B,C) Mouse placenta on gestational day (d) 7.5 (B) and d12.5 (C). (D-M) *Sirh7*/*Ldoc1* expression in the d7.5 (D), d8.5 (E), d9.5 (F,G), d12.5 (H,I), d15.5 (J,K) and d18.5 (L,M) placentas in wild-type mice. The boxed areas in F,H,J,L are magnified in G,I,K,M, respectively. The yellow lines enclose the area of the spongiotrophoblast layer. ch, chorion; epc, ectoplacental cone; gi, trophoblast giant cells (TGCs); sp, spongiotrophoblast layer; la, labyrinth layer; de, decidua; GlyT, glycogen trophoblast cells.

**Fig. 2.**
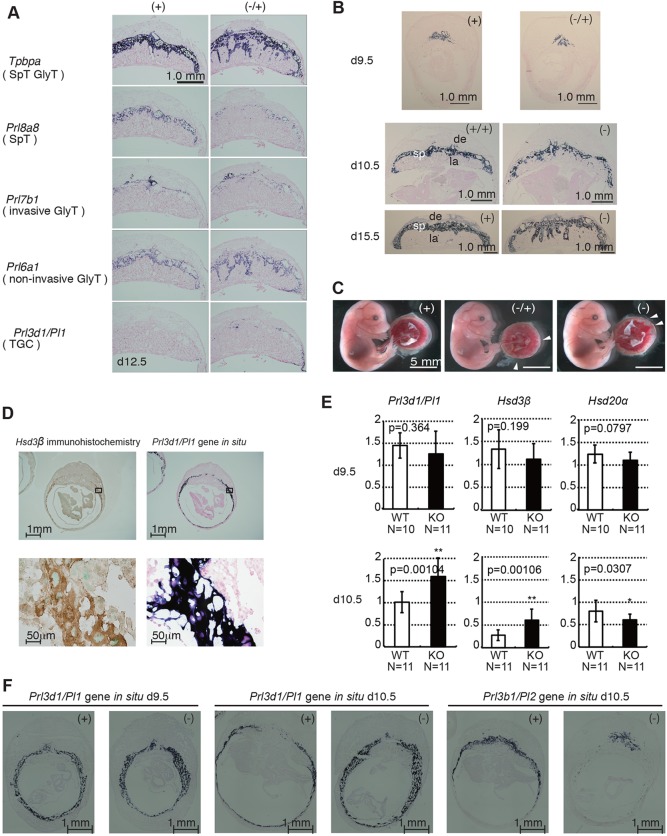
**Abnormal differentiation of *Sirh7/Ldoc1* KO placentas.** (A) Expression patterns of trophoblast lineage-restricted markers in the d12.5 placenta by RNA *in situ* hybridization. *Tpbpa*, *Prl8a8*, *Prl7b1*, *Prl6a1* and *Prl3d1* identify cells specific for spongiotrophoblast cells (SpT) and GlyT, SpT, invasive GlyT, non-invasive GlyT, and TGCs, respectively. Invasion of the *Prl6a1*-positive GlyTs took place in the labyrinth layer in only *Sirh7/Ldoc1* KO. There were fewer *Prl8a8*-positive SpTs in *Sirh7/Ldoc1* KO on d12.5. (B) Irregular spongiotrophoblast layer formation on d9.5, d10.5 and d15.5 (see A, *Tpbpa* on d12.5). (C) Fetuses and placentas on d15.5. The arrowheads indicate the regions where the labyrinth layers almost reached the maternal decidua. Despite the placental abnormality, *Sirh7/Ldoc1* KO fetuses looked normal (see also supplementary material Fig. S4). (D) Immunohistochemistry using an anti-Hsd3β antibody (left) and *in situ* hybridization for *Prl3d1* on d10.5 wild-type placenta (right). Higher magnifications of the boxed region of upper panels are shown in lower panels, respectively. (E) Relative expression levels of *Prl3d1*, *Hsd3b* and *Hsd20a* on d9.5 and d10.5 in each wild-type (+ or +/+) and KO (− or −/−) placenta (the expression level of β-actin was arbitrarily assigned a value of 1). White and black bars represent wild-type and KO placentas, respectively. Data are average±s.d. (**P<0.01, *P<0.005). (F) Expression patterns of the TGC markers *Prl3d1* (d9.5 and d10.5) and *Prl3b1* (d10.5) in wild-type and *Sirh7/Ldoc1* KO placenta by RNA *in situ* hybridization.

*Sirh7/Ldoc1* encodes a small Gag-like protein with an additional leucine-zipper motif at the N terminus (Fig. 1A), and is one of the eutherian-specific newly acquired genes from a sushi-ichi-related retrotransposon (supplementary material Fig. S1A,B) (Poulter and Butler, 1998; Brandt et al., 2005; Youngson et al., 2005; Ono et al., 2006; Volff, 2006). *Sirh7/Ldoc1* is predominantly expressed in the early stages of the placenta in a lineage-specific manner (Fig. 1B-M; supplementary material Fig. S2A): its high expression was observed in all of the placental cells, including TGCs and the ectoplacental cone cells (EPC) that subsequently produce spongiotrophoblast (SpTs), glycogen trophoblast (GlyTs) and various TGC subtypes on d9.5 (Fig. 1B,D-G). However, its expression gradually came to be restricted to GlyTs and then only remained in the so-called non-invasive type of GlyTs (*Prl6a1*-positive cells), and finally disappeared after the mid-stage of gestation (Fig. 1C,H-M and Fig. 2A).

*Sirh7/Ldoc1* is an X-linked gene and is subject to X chromosome inactivation: *Sirh7/Ldoc1* exhibits exclusive maternal expression in the placenta due to paternal X-chromosome inactivation, although it exhibits biallelic (more precisely, randomly monoallelic) expression in the embryo due to random X-chromosome inactivation (supplementary material Fig. S2B,C). In fact, not only structural but also functional abnormalities were observed in the KO placenta of *Sirh7/Ldoc1*^−/+^ female embryos with a maternally transmitted KO allele that was as seen in *Sirh7/Ldoc1*^−/−^ female and *Sirh7/Ldoc1^−^* male embryos, due to the lack of active maternal *Sirh7/Ldoc1* allele (Fig. 2A-C). The abnormality in TGCs was evident with two hormonal genes, *Prl3d1*/*Pl1* (encoding PL1) and *Prl3b1/Pl2* (encoding PL2) (Peng et al., 2002; Simmons et al., 2008). *Prl3d1* is the gene encoding PL1, one of the mouse prolactin-like proteins, and is used as a TGC-specific marker until midgestational stages. In the d10.5 placentas, the HSD3β protein was detected in TGCs by immunohistochemical analysis (Fig. 2D, left). Its expression site corresponded well with that of *Prl3d1* mRNA using *in situ* hybridization (Fig. 2D), supporting the previous result that *Hsd3b* expression was restricted in TGCs (Arensburg et al., 1999; Peng et al., 2002). *Prl3d1* and *Hsd3b* displayed significantly higher expression on d10.5 than the wild-type controls (∼1.5 and 2.0 fold; *P*=0.00104 and *P*=0.00106, respectively) whereas they were relatively lower on d9.5 (not statistically significant) (Fig. 2E, left and middle columns). Usually, the mRNA level of *Prl3d1* declines and that of *Prl3b1* mRNA increases after d10.5 in wild-type TGCs; however, this transition was apparently delayed in the *Sirh7/Ldoc1* KO, although at later stages *Prl3b1* expression was maintained at a normal level until delivery. It should be noted that *Prl3b1* expression is not specific to TGCs (Fig. 2F; supplementary material Fig. S3A). Despite these drastic changes, the number of TGCs was unchanged in the KOs on both d9.5 and d10.5, measured by counting *Prl3d1*-positive TGCs using a 10 μm center section of each conceptus; however, the thickness of the TGC layers looked increased on d10.5, as estimated by the areas of *Prl3d1*- and *Prl3b1-*producing cells detected with *in situ* hybridization experiments (Fig. 2F; supplementary material Fig. S3B). This may indicate that the development of KO embryos and placentas was slightly delayed in the early gestational stage. Because KO fetuses had normal weights on d12.5, d16.5 and d18.5, the catch-up phase of growth may occur during mid to late gestation, presumably due to a small increase in placental weight (supplementary material Fig. S4).

An irregular boundary between the spongiotrophoblast and labyrinth layers was also evident and was associated with a decrease in the number of SpTs (Fig. 2A; supplementary material Fig. S5). By RT-PCR, *Tpbpa* expression (a common marker for both SpTs and GlyTs) was shown to be decreased by a half to a third due to a decrease by a half to a third in the SpTs, as indicated by *Prl8a8* expression (a specific marker of SpTs). This took place despite a normal level of GlyTs, as indicated by *Pcdh12* expression (a specific marker of GlyTs), indicating the SpT/GlyT ratio in the spongiotrophoblast layer remained low from d12.5 to d15.5. SpTs are another important type of placental endocrine cells that also produce a variety of prolactin-like proteins (Ain et al., 2004; Bany and Cross, 2006; Simmons et al., 2008). We observed lower expression of SpT-specific *Prl3a1* and *Prl5a1* during these stages (supplementary material Fig. S3C). All of these results indicate that a variety of the placental cells were affected to some degree in the KO placentas.

### Pregnant *Sirh7/Ldoc1* KO females displayed delayed parturition associated with a low pup weaning rate

**Fig. 3.**
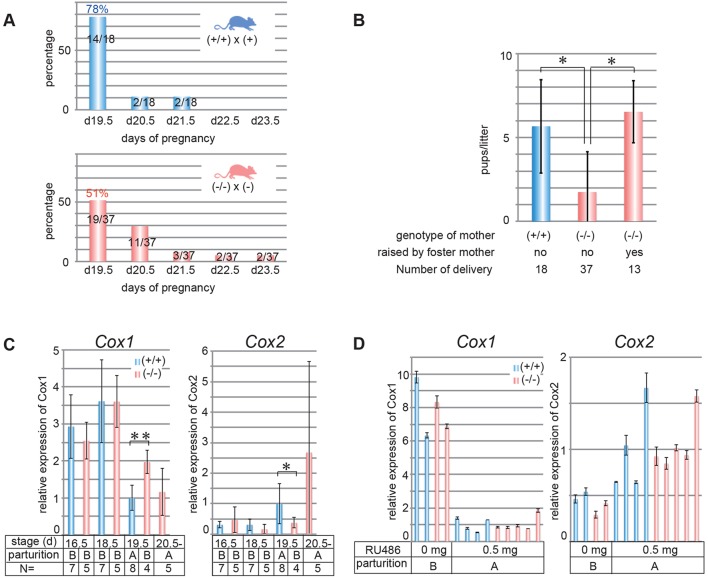
**Delayed parturition in *Sirh7/Ldoc1* female KOs.** (A) Delayed parturition in *Sirh7/Ldoc1*-null mice. Delivery days of +/+ and −/− females mated with + and − males, respectively [indicated as days post coitum (d)]. (B) Decreased weaning rates. Average litter size (mean±s.d.) for +/+ females mated with + males (blue) and −/− females mated with − males (red), with or without foster mothers (postpartum females) (*P<0.05). (C,D) Expression levels of *Cox1* (left) and *Cox2* (right) mRNAs without (C) and with RU486 injection (D) in +/+ (blue) and −/− (red) uteri on d16.5, d18.5, d19.5 and over d20.5 (before or after parturition) are shown (data are mean±s.d., **P<0.01, *P<0.05). (A,B) Mice after (A) and before (B) parturition. The average of +/+ uteri on d19.5 was defined as 1.

Another important phenotype of *Sirh7/Ldoc1* KO was that 49% of pregnant *Sirh7/Ldoc1* KO females displayed delayed parturition by ∼1-4 days, although 78% of the control mice normally delivered on d19.5 (Fig. 3A). The average number of their weaned pups was significantly lower than the controls (1.7 and 5.8 pups per litter, respectively, *P*<0.05) (Fig. 3B). Importantly, when foster mothers took care of the pups delivered from the KO mothers, the KO pups grew and weaned normally, indicating the KO pups themselves were not neonatal lethal (Fig. 3B). We did not see any clear difference in the number of weaned pups between the KO mothers exhibiting normal and delayed parturition, although both were low: 2.11±2.67 (d19.5, *n*=19), 2.00±2.41 (d20.5, *n*=11) and 0.43±1.13 (*n*=7), suggesting this is due to an intrinsic abnormality of KO mothers. However, when we examined the maternal behavior of KO mothers in terms of various criteria using virgin KO females, no abnormalities were observed (supplementary material Fig. S6). Therefore, the *Sirh7* KO mothers appear normal in terms of nursing behavior and it may be that the stress of parturition somehow gave rise to the abnormal maternal behavior in the case of *Sirh7/Ldoc1* KO.

**Fig. 4.**
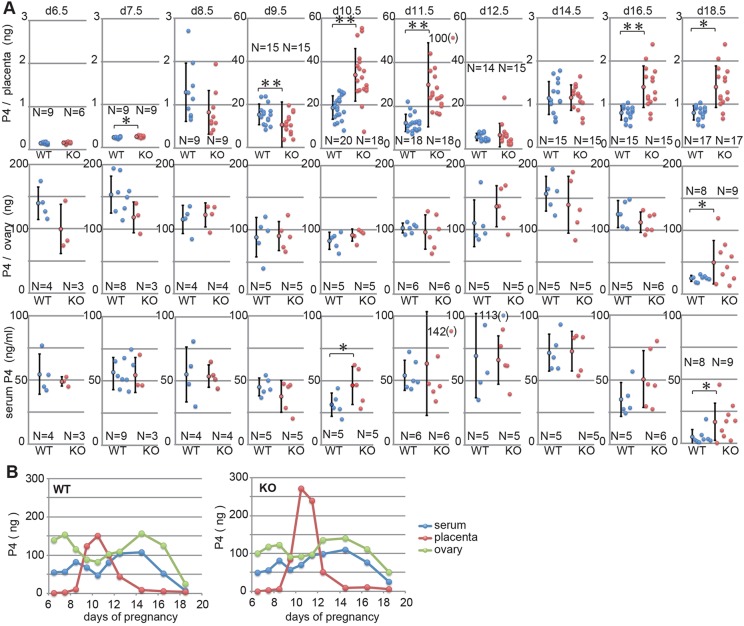
**Progesterone levels in the placenta, ovary and serum in wild-type and *Sirh7/Ldoc1* KO pregnant females.** (A) The amount of P4 in single placenta and two ovaries (both right and left), and the serum P4 concentration of each pregnant +/+ or −/− female (blue and red circles) on d6.5, d7.5, d8.5, d9.5, d10.5, d11.5, d12.5, d14.5, d16.5 and d18.5 are shown (data are mean±s.d., **P<0.01, *P<0.05). Each circle represents the P4 amount or P4 level of a single sample, except ovaries. (B) The average amounts of P4 in the placenta (red) and ovary (green) calculated as eight and two, respectively, and the serum P4 (blue) calculated as 1.5 ml per individual of each pregnant +/+ or −/− female on d6.5 to d18.5 are shown. At least three and six individuals were used for assay before and after d8.5, respectively.

We observed the higher serum P4 level on d18.5 (see Fig. 4A) in ∼50% of the KO females. In these KO females, the downregulation of cyclo-oxygenase 1 (*Cox1*) as well as the up-regulation of *Cox2* in the uterus, both of which trigger uterine contraction via prostagrandin F2α production (Tsuboi et al., 2000; Fortier et al., 2008), were also delayed (Fig. 3C). Subsequently, two experiments, RU486 (an antagonist of the P4 receptor, also known as an antagonist of glucocorticoid receptor signaling) administration (Fig. 3D and Table 1A) and ovariectomy (Table 1B), were carried out on d17.5 (Thorburn and Challis, 1979; Tsuboi et al., 2000; Piekorz et al., 2005). In both experiments, the KO females delivered the pups the next day. Normal reciprocal changes in *Cox1* and *Cox2* expression were also confirmed, demonstrating that the KO females responded to the rapid downregulation of serum P4 and thus started labor normally (Fig. 3D).

**Table 1.**
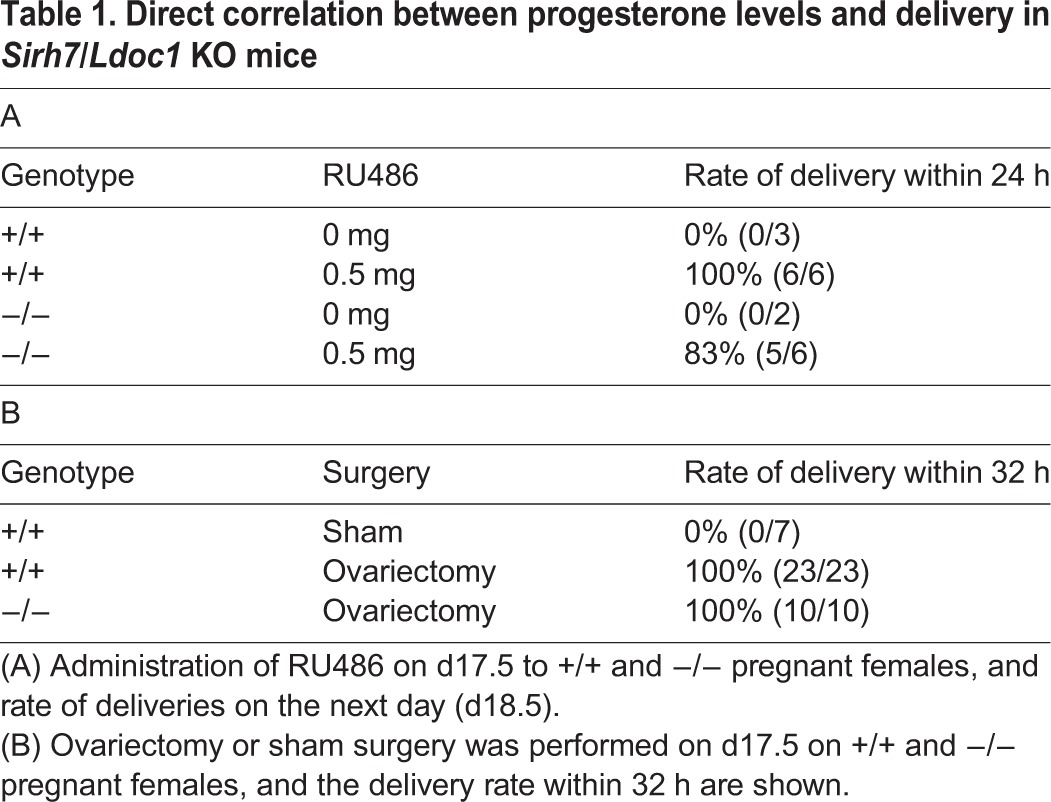
Direct correlation between progesterone levels and delivery in *Sirh7*/*Ldoc1* KO mice

### Reciprocal regulation of P4 production between ovary and placenta in mid-gestation and placental P4 overproduction in *Sirh7* KO mice

In order to address the relationship between the placental abnormality and the delayed parturition observed in *Sirh7* KO mice, we analyzed the P4 levels in the ovary, placenta and decidua from d6.5 to d18.5 (Fig. 4A,B, supplementary material Table S1) by improving the method of determining P4 in tissues and organs directly. The total P4 amount in the two ovaries per individual was well correlated with those of serum P4 (with a corresponding value of 1.5 ml serum per individual) throughout these periods; therefore, we judged this method to be highly reliable (Fig. 4A,B, left columns). This result confirms the previous view that the ovary plays a major role in the maintenance of serum P4 level during pregnancy (Malassine et al., 2003). However, we also detected the placental P4 during the period d9.5 to d12.5, coincident with the temporal reduction of ovarian as well as serum P4, while almost no P4 was detected in d6.5 and d7.5 samples that included both decidual and placental tissues. Importantly, the total amount (assuming eight placentas per individual) of placental P4 at its peak (d10.5) was almost equivalent to the maximum value of ovarian P4 on d14.5.

We also confirmed the reciprocal P4 production between the ovary and placenta in the KO mice (Fig. 4A,B, right). A striking feature of the KO placentas was that they produced approximately double the amount of P4 on d10.5 and d11.5 than the controls, although the amount was apparently lower on d9.5. It is known that the P4 level is controlled by the balance between its synthesis and degradation (Wiest et al., 1968; Piekorz et al., 2005). As shown in Fig. 2E (right column), the level of *Hsd20a* (*Akr1c18*, aldo-keto reductase family 1, member 18 – Mouse Genome Informatics), which encodes a P4 degradation enzyme (20α-hydroxysteroid dehydrogenase), was unaffected on d9.5, but slightly decreased on d10.5 (*P*=0.0307). Therefore, *Hsd3b* and *Hsd20a* were expressed in both the d9.5 and d10.5 placentas, suggesting that the actual P4 level is determined by the balance of these two enzymes in the mid-gestation placenta, as in the case of the ovary (Wiest et al., 1968; Piekorz et al., 2005). Interestingly, the placental P4 production pattern in the KO placenta itself looked slightly delayed, lower on d9.5 and higher on d10.5 and d11.5, and even on d11.5 its level was higher than the peak level of the control placenta on d10.5 (Fig. 4A,B), then the patterns of ovarian P4 production and serum P4 levels after d12.5 also seemed to be slightly delayed, leading to the higher serum P4 levels that remained in the late gestation period in half of the KO mice.

## DISCUSSION

In this work, we demonstrated that *Sirh7/Ldoc1* KO mice exhibited several abnormalities, such as structural and endocrinological defects in the placenta, delayed parturition and a low weaning rate. Each of these is a very important issue in mouse development and reproduction, but it is difficult to directly correlate these phenotypes with each other in terms of the regulatory control exerted between the ovary and placenta. The results here show that delayed and/or abnormal differentiation/maturation of TGCs at the early stages of gestation led to the excess placental P4 and/or PL1 on d10.5, delayed downregulation of the former on d11.5 and the delayed transition from PL1 to PL2 in KO mice. Therefore, it is possible that these hormonal changes are related to the delayed parturition and somehow shift the ovarian P4 production backwards in the second-half of pregnancy, leading to a higher serum P4 concentration in the KO females. Alternatively, the higher placental P4 level on d18.5 may account for the delay via directly regulating local P4 levels within the uterus, although its level was only 1/20 of the peak (Fig. 4A,B). As a reduction of *Prl3a1* and *Prl5a1* production was observed in the KO placenta (supplementary material Fig. S3C), it also seems likely that such late stage-specific prolactin-like proteins produced from specific SpTs play an important role as a delivery signal, although no such activity has been reported to date.

From the result of the foster mother experiment, it is apparent that the low weaning rate of the KO pups was due to a maternal defect. However, it is difficult to conclude whether it is an intrinsic problem in KO mothers or a problem associated with the stress of parturition. The latter seems probable, because that much stress in labor could well impede normal maternal behavior in some cases. It might be difficult to identify such maternal problems from behavioral tests using virgin KO females. Alternatively, it is also possible that that *Sirh7/Ldoc1* is somehow involved in the maternal behavior via an effect on brain function. As already mentioned, the predominant expression of *Sirh7/Ldoc1* occurred in the placenta, but it was also detected in cerebellum and cerebrum in adults, although at 1/40 and 1/500 of the level in the placenta, respectively (supplementary material Fig. S2A). Therefore, we cannot completely exclude this possibility at present. All these issues will need to be addressed in the future by generating placenta- and/or brain-specific KO mice, as well as elucidating the biochemical function of Sirh7/Ldoc1 protein.

In the course of this study, we substantially improved the P4 assay by using mass spectrography for the direct detection of P4 in several tissues and organs. This enables the identification of the organ that produces P4, as well as when and to what extent during the course of pregnancy. As a result, it was clearly demonstrated that mouse placentas produce P4 in mid-gestation when a temporal reduction of serum P4 level occurs due to a shift from the corpus luteum of pseudopregnancy to pregnancy. What, then, is the biological role of the placental P4? P4 plays an essential role in the protection of the conceptus from maternal immunity as ovariectomy at anytime during the course of pregnancy causes rapid abortion that can be rescued completely by intraperitoneal P4 injection (Deanesly, 1973; Clemens et al., 1979; Siiteri and Stites, 1982; Szekeres-Bartho et al., 2009). It is known that the serum P4 concentration exhibits temporal downregulation of uncertain cause around d10.5. As the ovary stops P4 production intrinsically on d10.5 in pseudopregnant mice (supplementary material Fig. S7), this strongly suggests that the placental P4 compensates for the reduced serum P4 concentration in the pregnant mice in a site-specific manner (Fig. 4A,B). This also suggests that the placenta is required for the recovery of ovarian P4 production in late gestation. Although analysis of the placenta-specific *Hsd3b* knockout mouse will be required to clarify these issues, these results provided new insights on the role of mouse placental P4 in the maintenance of pregnancy and in the determination of parturition timing during mid- and late gestation, respectively. We believe that this method opens a new chapter in reproductive biology because information on the precise timing and sites of P4 production will help elucidate the regulation of pregnancy, not only in eutherians but also in marsupials, as well as reproduction in oviparous monotremes and non-mammalian vertebrates, such as birds, reptiles and amphibians (Tyndale-Biscoe and Renfree, 1987; Etches and Petitte, 1990).

Pituitary prolactin is the major hormone that supports ovarian P4 production in the first half of pregnancy; PL1 and, subsequently, PL2 from the placenta take over this role in the last half (Malassine et al., 2003). In 1971, Bartke hypothesized that the mouse placenta produces prolactin-like hormone before it produces the full luteotrophic complex, because pregnancy was maintained when the source of pituitary prolactin was removed specifically on day 9 or 10 in dwarf mice with hereditary prolactin deficiency with a normal mouse pituitary implanted under the kidney capsule (Bartke, 1971). Together with the these previous works, our own results showed that it is placental P4 that plays the protective role and is absolutely required for survival of the conceptus in this specific period.

In eutherians, P4 production in the corpus luteum is regulated by pituitary and/or placenta whereas it is autonomous in marsupials and monotremes (Tyndale-Biscoe and Renfree, 1987). It will therefore be of interest to elucidate how *Sirh7/Ldoc1* functions in these processes at the molecular level because *Sirh7/Ldoc1* is a eutherian-specific gene derived from a sushi-ichi-related retrotransposon (supplementary material Fig. S1A,B). Our results demonstrate that *Sirh7/Ldoc1* is essential for placental cell differentiation/maturation and thus intrinsically related to the regulation of placental hormones. This means that the shape and/or composition determine the functions of the placenta, including endocrine regulation and nutrient support. Its deletion led to the overproduction of placental P4 and PL1, and the delayed parturition associated with a low pup weaning rate, suggesting that the endocrine function of the placenta also plays an important role in the regulation of the timing of parturition. We have previously reported that *Peg10* and *Peg11*, two other retrotransposon-derived genes, play essential roles in the formation and maintenance of the placenta, respectively (Ono et al., 2006; Kagami et al., 2008; Sekita et al., 2008). Together with the previous results, the present findings provide insight into the impact of the LTR retrotransposon-derived SIRH family of genes on the evolution of the mammalian viviparous system.

## MATERIALS AND METHODS

### Animals

All animal studies were conducted in accordance with the animal care guidelines approved by the committee of Tokyo Medical Dental University and Tokai University. Animals were allowed access to a standard chow diet and water *ad libitum*, and were housed in a pathogen-free barrier facility with a 12 h light:12 h dark cycle. C57BL/6J (B6) mice were used throughout study as controls. To produce *Sirh7/Ldoc1* KO mice, we obtained 8.9 kb (nucleotides 129,912-138,850; AL672025) and 2.4 kb (nucleotides 140,484-142,859; AL672025) genomic fragments by screening the 129SvJ lambda genomic library (Stratagene). We used these fragments as the right- and left-arm sequences of a construct in which the *Sirh7/Ldoc1* gene was replaced with the neomycin resistance gene. After a 1-week incubation under G418 selection followed by electroporation of the linearized DNA into embryonic stem cells (ESCs) (CCE) of 129/Sv/Ev mouse origin. Homologous recombination events were screened by Southern blotting. ESC culture and blastocyst injection were performed as described previously (Ono et al., 2006). A total of 8 *Sirh7/Ldoc1*-targeted ESC clones that resulted from homologous recombination of the construct were identified, and chimeric males were generated from two of these clones following blastocyst injection. Germline transmission of the knockout allele was confirmed in three chimeric males from each clone. *Sirh7/Ldoc1* KO mice were backcrossed to B6 more than 10 generations. To delete the neomycin resistance gene, we carried out *in vitro* fertilization using normal B6 sperm and eggs from *Sirh7/Ldoc1* KO with *Neo* females or sperm from *Sirh7/Ldoc1* KO with *Neo* male and normal B6 eggs and injected the Cre recombinase expression vector (pCAGGS-Cre). The *Sirh7/Ldoc1* KO female mice, which inherited the KO allele from their mothers are represented as *Sirh7/Ldoc1* (−/+), whereas the *Sirh7/Ldoc1* KO mice that inherited the KO allele from their fathers are shown as *Sirh7/Ldoc1* (+/−). Male *Sirh7/Ldoc1* KO mice that have only one X chromosome are represented as *Sirh7/Ldoc1* (−). *Sirh7/Ldoc1* KO mice were crossed with B6 for more than 10 generations. In timed pregnancy studies, age-matched *Sirh7/Ldoc1* (−/−), *Sirh7/Ldoc1* (−/+) and *Sirh7/Ldoc1* (+/+) mice were monitored for the presence of a vaginal plug on 0.5 day postcoitum.

### Measurement of progesterone levels

To measure the concentration of P4 in various tissues, the serum, placenta (including decidual cells) and ovaries were collected from B6 mice. Blood was collected from the abdominal aorta. The blood was left at room temperature for 30 min and centrifuged at 2000 ***g*** for 15 min at 4°C, then the supernatant was collected. Before analysis, internal standard P4 and ethyl acetate were added and mixed thoroughly. Placentas (∼d6.5-18.5) and ovaries (∼d6.5-18.5) were dissected out with scissors, rapidly frozen in liquid nitrogen and kept frozen at −80°C until use. These samples were homogenized with ULTRA-TURRAX (IKA) and then the internal standard P4 and ethyl acetate were added and further homogenized on ice. After centrifugation, the supernatant (ethyl acetate extracts) was isolated and separated with ion-chromatography (Oasis Max, Waters). Progesterone was separated by further centrifugation/evaporation and normal phase chromatography (Intertsep SI, GL Sciences) and applied to LC-MS/MS. The progesterone concentration was measured at ASUKA Pharma Medical (Kawasaki, Japan). It is essential that the samples are subjected to P4 assay as soon as the sample preparation is finished. Otherwise, we recommend a short heat treatment using a microwave oven for ∼3-4 min before homogenizing the samples in order to obtain stable results (see supplementary material Table S1). *Sirh7/Ldoc1*^−/−^ mice were analyzed by the same method.

### PCR for genotyping

DNA samples for PCR were prepared from the embryos and embryonic yolk sacs using ISOGEN (Nippon Gene) and the tail using a DNeasy Blood and Tissue kit (Qiagen). A mixture containing 1× ExTaq buffer (TaKaRa), 2.5 mM dNTP mixture, primers and 2.5 U of ExTaq (TaKaRa) was subjected to 32 PCR cycles of 96°C for 15 s, 65°C for 30 s and 72°C for 30 s in a Bio-Rad C1000 system. The PCR primers used can be found in supplementary material Table S2.

### RNA isolation and RT-PCR

Total RNA samples were prepared from the adult tissues, embryos and placentas of mice at various stages using ISOGEN (Nippon Gene). The cDNA was synthesized from 1 μg of total RNA using Superscript III reverse transcriptase (Life Technologies) according to the manufacturer's protocol. RT-PCR was performed as previously described (Ono et al., 2006).

### Allelic expression analysis

DNA polymorphism between JF1 and B6 were detected using restriction fragment length polymorphism (RFLP) and single site polymorphism (SSP) analyses. Genomic DNA and total RNA were prepared from the embryos and placentas of the F1 of (B6×JF1) and (JF1×B6) mice. The RT-PCR methods were as described previously (Ono et al., 2006) and primer sequences of *Sirh7*/*Ldoc1* can be found in supplementary material Table S2. For RFLP analysis, *Sirh7/Ldoc1* was digested with *Hpy*CH4IV.

### *In situ* hybridization

The implantation sites (d7.5-10.5) and placentas (d12.5-18.5) were dissected in cold phosphate buffered saline (PBS) and fixed overnight in 4% paraformaldehyde (PFA) in PBS at 4°C and tissues were subjected to graded sucrose solutions (10% in PBS and 25% in PBS) before being embedded in OCT (Tissue Tek). Ten micron sections were cut on a cryostat (MICROM), mounted on glass Super frost micro slides (Matsunami), and stored at % 80°C. *In situ* hybridization with *Sirh7/Ldoc1*, *Tpbp*, *Pcdh12*, *Prl8a8*, *Prl3d1/Pl1*, *Prl3b1/Pl2*, *Prl7b1* and *Prl6a1* antisense probes was performed on tissue sections as previously reported (Simmons et al., 2008). The nucleotide sequences that were used for *in situ* probes can be found in supplementary material Table S2.

### Quantitative RT-PCR

The PCR products were quantified using Applied Biosystems 7500 Real-time PCR system with Fast SYBR Green Master Mix according to the manufacturer's instructions. Each reaction was carried out as previously described (Ono et al., 2006). The primer sequences can be found in supplementary material Table S2.

### RU486 treatment

Injections of mineral oil (control) or RU486 (mifepristone, Sigma-Aldrich), an antagonist of the progesterone receptor, were administered subcutaneously (0.5 mg in mineral oil) to pregnant mice on d17.5 or +/+ and −/− mice were ovariectomized on d17.5. Each uterus was collected on the following day (d18.5) for relative mRNA determination (mean±s.e.m.). All the pregnant females receiving RU486 treatment delivered pups on d18.5 regardless of genotype, whereas no control females did, as expected.

### Ovariectomy

After anesthetization, the +/+ and −/− mice were ovariectomized (OVX) or sham-operated (Sham). Ovaries were bilaterally removed by incision. The uterine horns were tied and the uterus was left intact. The abdominal wall was then sutured. After surgery, mice were maintained under good conditions and allowed to recover. In the sham procedure, animals were anesthetized and the abdominal wall was opened in a similar manner to that used for the OVX mice; the ovaries were exteriorized to create similar stress, but they were not removed.

### Statistical analysis

Results are presented as mean±s.d. Differences between two groups were analyzed with the Student's *t*-test. *P* values of less than 0.05 was considered statistically significant.

### Comparative genomics analysis

Identification of the *Sirh7/Ldoc1* orthologous genomic region was performed using UCSC genome browser (http://genome.ucsc.edu/cgi-bin/hgGateway) against mammalian genomes using human *SIRH7/LDOC1* as a query (GenBank ID. NP_036449). Alignments were obtained using the VISTA Web server (http://www-gsd.lbl.gov/VISTA). *SOX3-SLITRK4* syntenic regions of several species identified above were aligned using the default setting (>70% identity and >100 bp in length) of mVISTA with the Shuffle-LAGAN program. The sequences of human *SOX3-SLITRK4* syntenic region (139,500K-143,500K bp *Homo sapiens* chromosome X genomic contig, Annotation Release 105), mouse *Sox3-Slitrk4* syntenic region (60,500K-64,500K bp *Mus musculus* strain C57BL/6J chromosome X genomic contig, Annotation Release 103), sheep *Sox3-Slitrk4* syntenic region (c46M-50M bp *Ovis aries* chromosome X genomic contig, Annotation Release 100), opossum *Sox3-Slitrk4* syntenic region (46M-50M bp *Monodelphis domestica* chromosome X genomic contig, Build2.2) and chicken *SOX3-SLITRK4* syntenic region (8M-12M bp *Gallus gallus* chromosome 4 genomic contig, Annotation Release 103) were extracted from NCBI (http://www.ncbi.nlm.nih.gov/).

## Supplementary Material

Supplementary Material
