# Supplementary material for: Sirh7/Ldoc1 knockout mice exhibit placental P4 overproduction and delayed parturition
Source: Development. 2014 Dec 15;141(24):4763–71. doi: 10.1242/dev.114520 (PMC4299276; doi:10.1242/dev.114520)
Supplement: Supplementary Material [file supp_141_24_4763__index.html]

Supplementary Material 

# *Sirh7/Ldoc1* knockout mice exhibit placental P4 overproduction and delayed parturition

## DEV114520 Supplementary Material

**Files in this Data Supplement:**

- Supplementary Material
