# Supplementary material for: Sirh7/Ldoc1 knockout mice exhibit placental P4 overproduction and delayed parturition
Source: Development. 2014 Dec 15;141(24):4763–71. doi: 10.1242/dev.114520 (PMC4299276; doi:10.1242/dev.114520)
Supplement: Supplementary Material [file supp_141.24.4763_DEV114520supp.pdf]

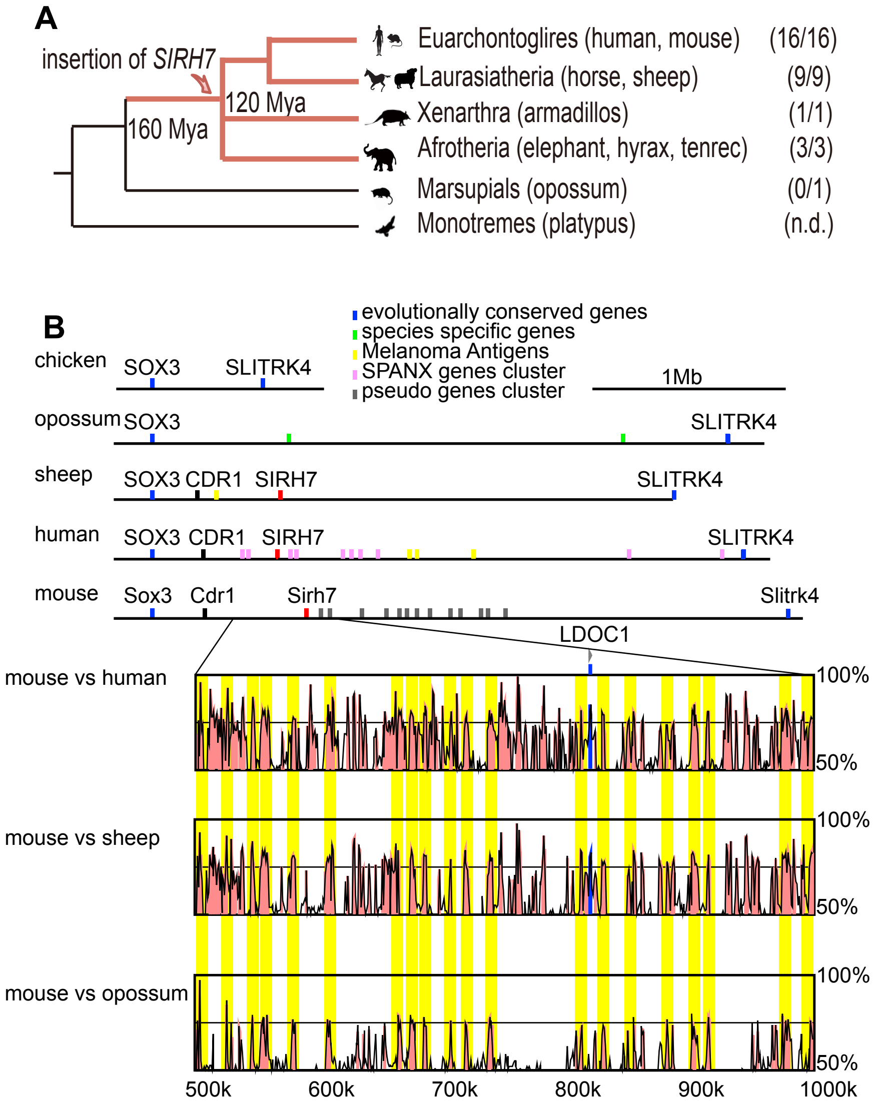

Fig. S1. *Sirh7/Ldoc1* conservation in eutherian mammals.
A *Sirh7/Ldoc1* was confirmed in 29 eutherian species covering all four major groups, such as euarchontoglires, laurasiatheria, xenarthra and afrotheria, but was absent from marsupials, indicating that the insertion of *Sirh7/Ldoc1* occurred in a common eutherian ancestor. The numbers of species that are confirmed to have *Sirh7/Ldoc1* are shown in parentheses. **B** *SIRH7/LDOC1* is conserved in the orthologous chromosomal region between *SOX3* and *SLITRK4* only in the eutherians but absent in the marsupials (upper column) although several evolutionary conserved sequences between the marsupials and eutherians are present around *SIRH7/LDOC1* (lower column, vertical yellow bars). No *SIRH7/LDOC1* orthologue was also detected in two Australian marsupial and one monotreme species, the tammar wallaby, Tasmanian devil and platypus. However, there are still some gaps between *SOX3* and *SLITRK4* in their genome sequences, therefore, we did not count these.

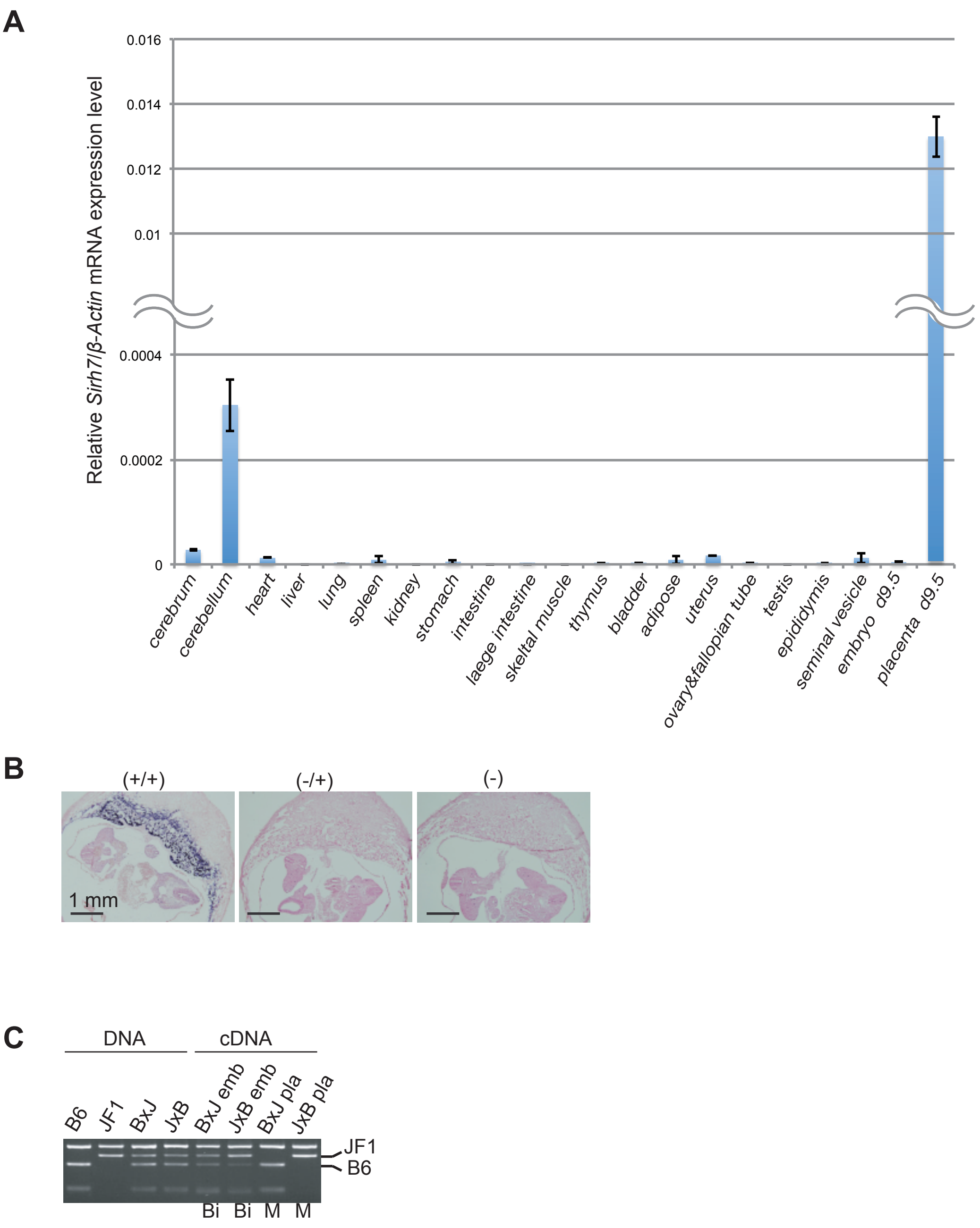

Fig. S2. *Sirh7/Ldoc1* expression in adult tissues and organs and maternal specific *Sirh7/Ldoc1* expression in the placenta.
A Low levels of *Sirh7/Ldoc1* expression were observed in the cerebellum and cerebrum in adults (19w) compared with the highest expression in the d9.5 placenta. The relative expression levels of *Sirh7/β-Actin* mRNA in 19 adult organs, embryos (d9.5) and placenta (d9.5) were shown. Note that the different magnification percentages are used between the lower and upper parts so as to easily compare the samples in lower expression levels. **B** *In situ* hybridization using a *Sirh7/Ldoc1* antisense probe in (+/+), (-/+) females and (-) male placentas on d10.5. As *Sirh7/Ldoc1* is an X-linked gene and subject to X chromosome inactivation, maternal transmission of a *Sirh7/Ldoc1* KO allele in the X chromosome induces null expression in both females and males. **C** Restriction fragment length polymorphism (RFLP) analysis of *Sirh7/Ldoc1* was carried out between C57BL6/J and JF1 using d9.5 embryos and placentas. *Sirh7/Ldoc1* exhibits exclusive maternal expression in the placenta due to paternal X chromosome inactivation, while it is biallelically expressed in the embryo due to random X chromosome inactivation. Bi: Biallelic expression, M: Maternal expression.

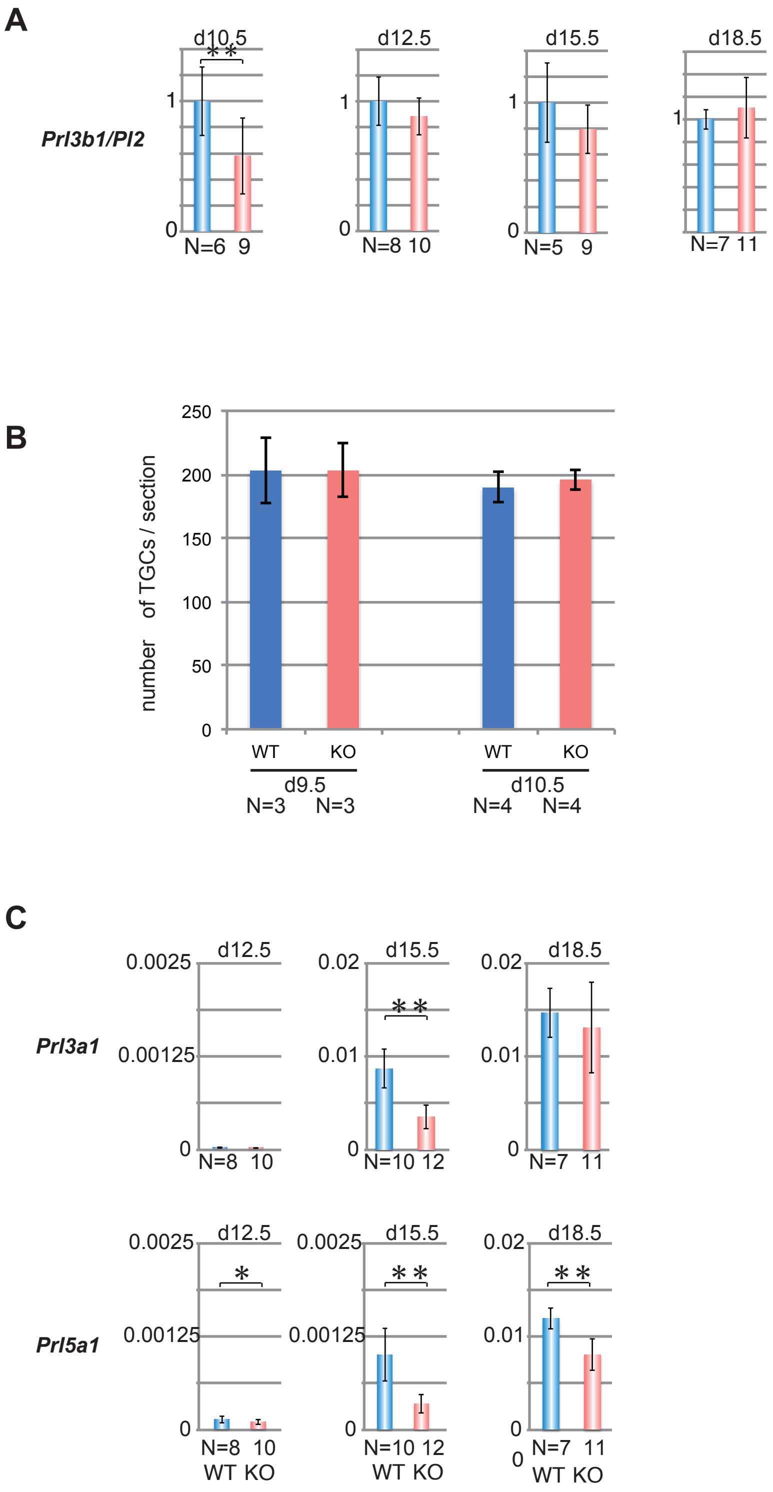

Fig. S3. Abnormal differentiation of *Sirh7/Ldoc1* KO placentas.
A Relative expression levels of *Prl3b1/Pl2* on d10.5, d12.5, d16.5 and d18.5 in WT (+ or +/+) and KO (- or -/+) placentas, normalized with *β-Actin*. Data are shown as the average ± s. d. (**: $p < 0.01$ and *: $p < 0.05$). **B** The total number of TGCs by counting nuclei stained with DAPI using the 10 μm center sections next to those used for *Prl3d1/Pl1 in situ* hybridization experiment (Fig. 2F) to confirm the position of *Prl3d1/Pl1* expressing TGCs. **C** Relative expression levels of *Prl3a1* and *Prl5a1* on d12.5, d16.5 and d18.5 in WT (+ or +/+) and KO (- or -/+) placentas, normalized with *β-Actin*. Data are shown as the average ± s. d. (**: $p < 0.01$ and *: $p < 0.05$).

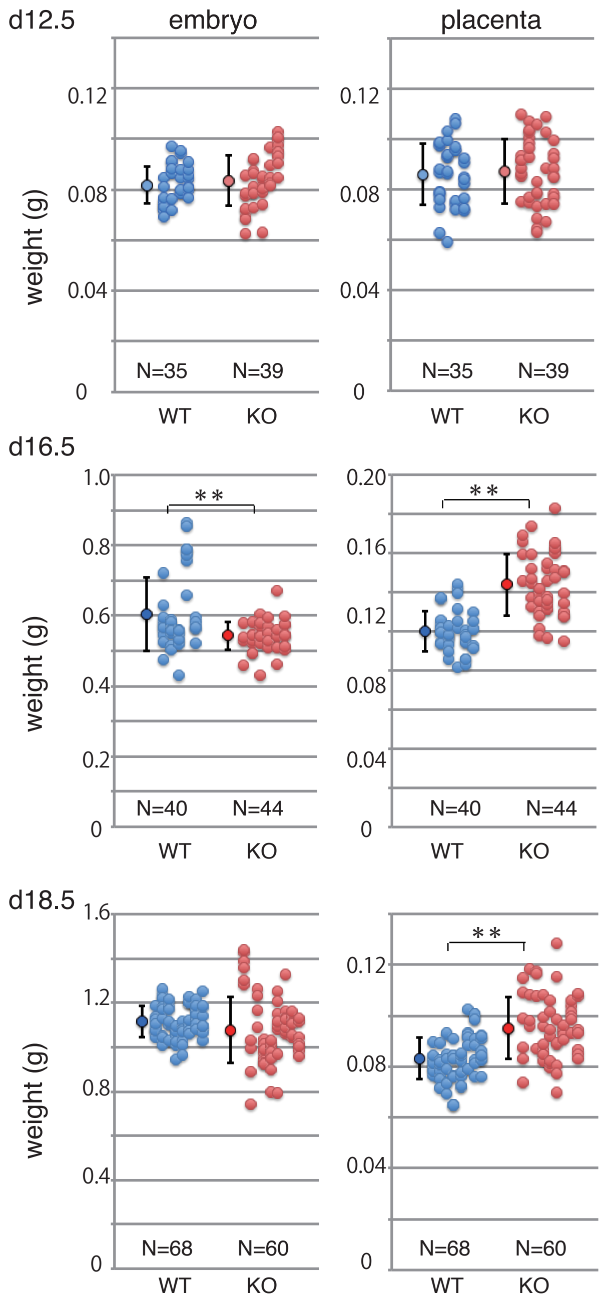

Fig. S4. Fetal and placental weights during development.
WT and KO littermates at d12.5, d16.5 and d18.5 were measured. Each blue or red circle represents the weight of a single WT or KO sample, respectively, and a black-edged blue or red circle represents the average weight. Data are shown as the average ± s. d. (**: $p < 0.01$). Embryos and placentas in the same litter are presented in a line.

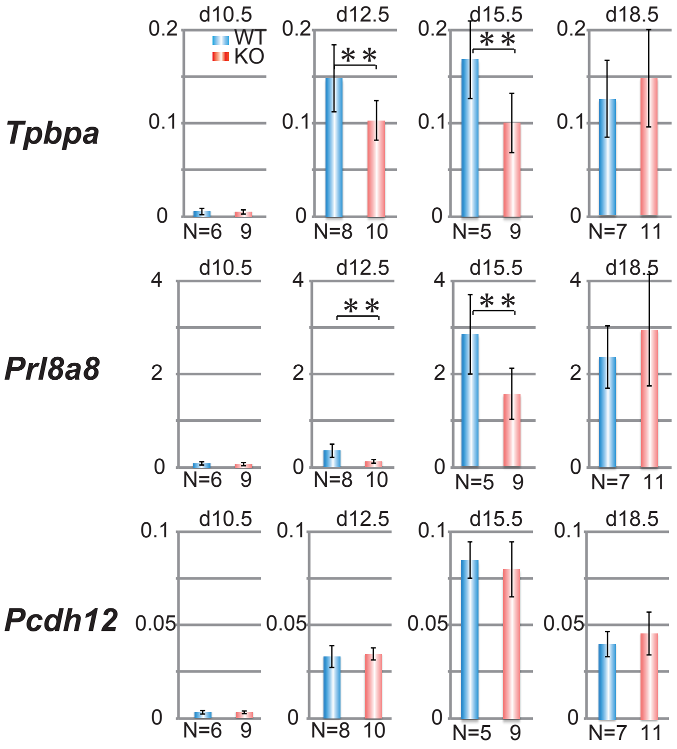

Fig. S5. Decreased number of SpTs in *Sirh7/Ldoc1* KO placenta.
Relative expression levels of *Tpbpa*, *Prl8a8* and *Pcdh12* on d10.5, d12.5, d16.5 and d18.5 in WT (+ or +/+) and KO (- or -/+) placentas, normalized with *β-Actin*, were shown. Expression levels of both *Tpbpa* (SpT and GlyT) and *Prl8a8* (SpT) were decreased 2/3 and 1/3~1/2 fold, respectively, while *Pcdh12* (GlyT) expression was normal, indicating the SpT/GlyT ratio in the spongiotrophoblast layer remained low from d12.5 to d15.5. Data are shown as the average ± s. d. (**: $p < 0.01$).

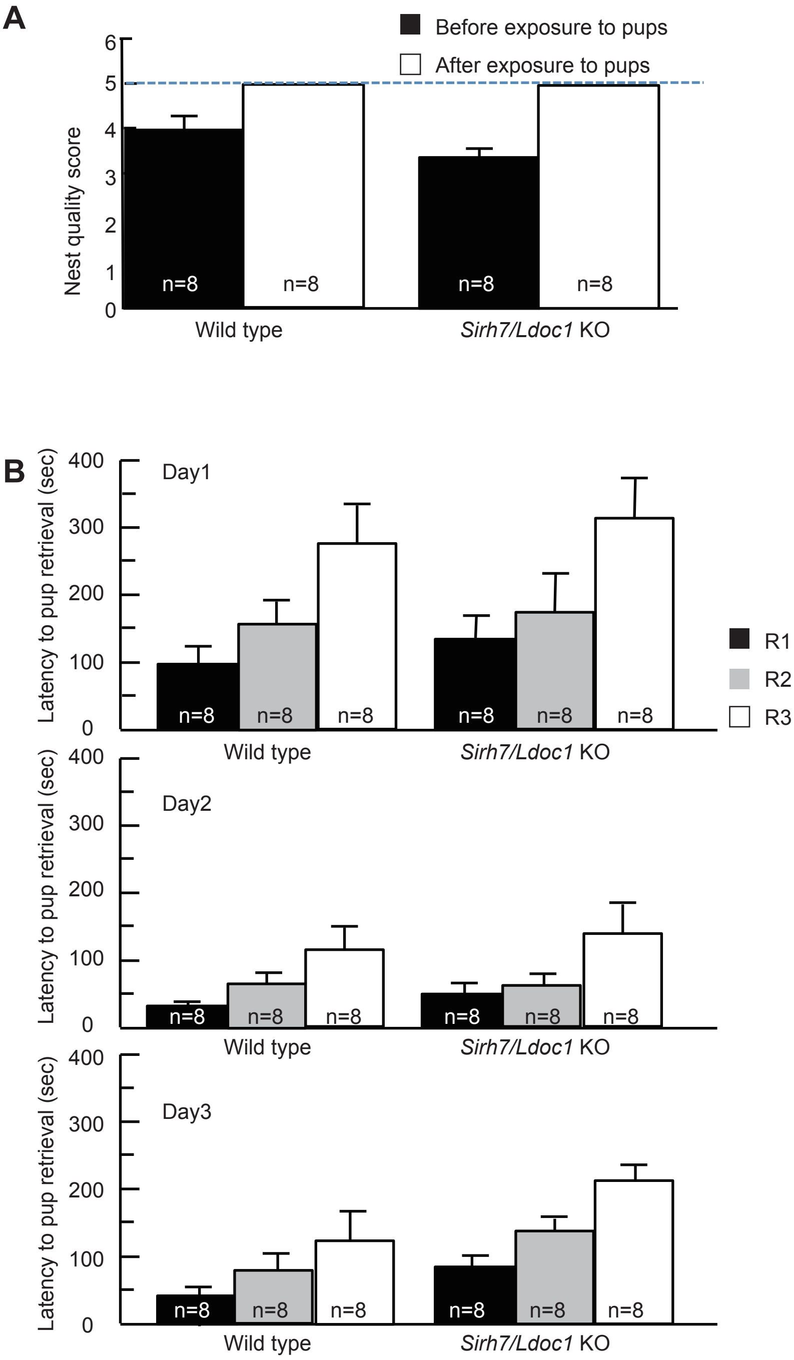

Fig. S6. Normal maternal behavior of *Sirh7/Ldoc1* KO mice.
A Nest evaluation. Nest quality scores (1-5) of the (+/+) and (-/-) females before (black bars) and after (white bars) exposure to pups were measured using 11w old virgin mice. They were provided with a piece of packed cotton pulp (5 cm x 7 cm) as nest material in addition to the usual wooden chips. Nest quality scores 1: Nest material not noticeably touched (>90% intact), 2: Nest material partially torn up (50–90% remaining intact), 3: Mostly shredded (10–50% intact) but not gathered into a nest, 4: An identifiable but flat nest: <10% Nest material intact, but incomplete walls, 5: A (near) perfect nest with walls built up to form a crater: <10% Nest material intact.
B Pup retrieval assay using virgin females. Virgin (+/+) and (-/-) mice were exposed to three WT young pups for 10 min on 3 consecutive days (Day 1: black bars, Day 2: gray bars, Day 3: white bars) and measured the latency to retrieval. All of the virgin females responded to the pups and carried them to nests. The latency to pup retrieval was not different between the (+/+) and (-/-) virgin females on Day 1 and Day 2. Only on Day 3 was latency slightly longer. R1: Latency to first pups, R2: Latency to second pups, R3: Latency to third pups.

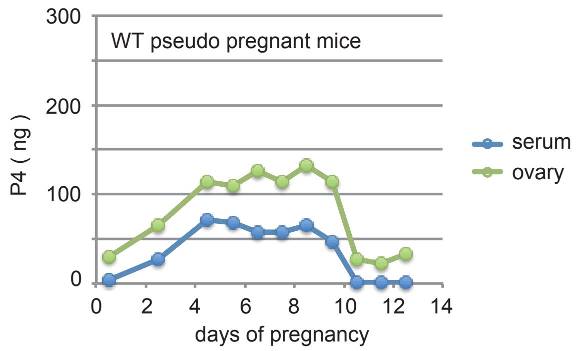

Fig. S7. Progesterone (P4) levels in the ovary and serum in WT pseudo pregnant mice.
The average amounts of P4 in ovary (green) calculated as two and the serum P4 (blue) calculated as 1.5 ml per individual of each pregnant female on d0.5 to d12.5 are shown. Two to three individuals were used for assay. It should be noted that the levels of serum and ovarian P4 were almost equivalent to those of the normal pregnant mice until d9.5, confirming the shift from the corpus luteum of pseudopregnancy to pregnancy occurs around d10.5.

Table S1. Estimated placental P4 levels with or without heat treatment.
Samples: Day 12.5 placentas (B6)

Heat treatment	Recovery ratio of I.S. (%)	Estimated P4 amount (ng/placenta)
-	90.5	5.75
-	57.6	3.00
-	24.4	5.40
-	21.9	4.30
-	4.0	7.45
-	3.7	6.77
-	1.4	10.15
done	109.7	1.64
done	101.2	4.97
done	97.9	5.56
done	97.2	1.53
done	86.4	2.97
done	86.0	2.02
done	71.7	2.78

*** Without heat treatment, severe degradation of internal standard P4 sometimes occurred, leading to higher estimated values of P4 amount.**

Supplementary Table 2. PCR primers

primer name	primer type	sequence
Sirh7/Ldoc1 forward	PCR for genotyping	5'-TGA AGGCTGGAGACCGC-3'
Sirh7/Ldoc1 (WT allele) reverse1	PCR for genotyping	5'- ATGGTTAACTGCTTGGACGG-3'
Sirh7/Ldoc1 (KO allele) reverse2	PCR for genotyping	5'-CGCAATGGC CAGTACTAGTG-3'
Sirh7/Ldoc1 forward	Allelic expression analysis and Quantitative RT-PCR	5'-ACATCCACTGGAACTCAGGC-3'
Sirh7/Ldoc1 reverse	Allelic expression analysis and Quantitative RT-PCR	5'-CATGGA CACATCTGTGAGGC-3'
Sirh7/Ldoc1 forward	*in situ* hybridization analysis	5'-GGCTTCTGTACAGGTAGAGCAGAC-3'
Sirh7/Ldoc1 reverse	*in situ* hybridization analysis	5'-AGATCTAGTTTTGCAGTGCTTGATATTTATTG-3'
Tpbpa forward	*in situ* hybridization analysis	5'-AAGTTAGGCAACGAGCGAAA-3'
Tpbpa reverse	*in situ* hybridization analysis	5'-TATGGGAGAGTTTGTGGGGA-3'
Prl8a8 forward	*in situ* hybridization analysis	5'- AACTCCGATGAAGCAGATCTC-3'
Prl8a8 reverse	*in situ* hybridization analysis	5'-TTTGTGGAGCGTCTCCAAAG-3'
Prl7b1 forward	*in situ* hybridization analysis	5'- GACACCAGTTTAGCAGCC TTT-3'
Prl7b1 reverse	*in situ* hybridization analysis	5'-CTGAGTCTTGGCACATGTAACC-3'
Prl6a1 forward	*in situ* hybridization analysis	5'-CCACAGACGTGGTCATTTCCC-3'
Prl6a1 reverse	*in situ* hybridization analysis	5'-ACGTGAATCCTTGACCAAGCAC-3'
Prl3d1/Pl1 forward	*in situ* hybridization analysis	5'-TTGGCCGCAGATGTGTATAG-3'
Prl3d1/Pl1 reverse	*in situ* hybridization analysis	5'-AACTGAGGAGGGGAAAGCAT-3'
Prl3b1/Pl2 forward	*in situ* hybridization analysis	5'-TCGATTACCCACTGAAAGCC-3'
Prl3b1/Pl2 reverse	*in situ* hybridization analysis	5'-AGAAAGGCACCAAAGAAGGG-3'
β-actin forward	Quantitative RT-PCR	5'-AAGTGTGACGTTGACATCCG-3'
β-actin reverse	Quantitative RT-PCR	5'- GATCCACATCTGCTGGAAGG-3'
Prl3d1/Pl1 forward	Quantitative RT-PCR	5'-GGC AGAAACCTTGTAATTCTGG-3'
Prl3d1/Pl1 reverse	Quantitative RT-PCR	5'-GGGCACTCAACATTCGTTC-3'
Hsd3β forward	Quantitative RT-PCR	5'-CAGACCATCCTAGATGTCAATCTG-3'
Hsd3β reverse	Quantitative RT-PCR	5'- ACTGCCTTCTCAGCCATC-3'
Hsd20α forward	Quantitative RT-PCR	5'-ACCAGCTGGACCGTGGGATT-3'
Hsd20α reverse	Quantitative RT-PCR	5'-TTGGAGGCGGTGTGTCGAGT-3'
Cox1 forward	Quantitative RT-PCR	5'-ACTGGTCTGCCTCAACACCA-3'
Cox1 reverse	Quantitative RT-PCR	5'-GCCTCAAACTCCCAGAGATCCA-3'
Cox2 forward	Quantitative RT-PCR	5'-AAGCGAGGACCTGGGTTCA-3'
Cox2 reverse	Quantitative RT-PCR	5'-AAGGCGCAGTTTATGTTGTCTGT-3'
Prl3b1/Pl2 forward	Quantitative RT-PCR	5'-GGGCTTCTGGAAGGACTGA-3'
Prl3b1/Pl2 reverse	Quantitative RT-PCR	5'-CACGAGGGACCTTCTAAGAGAC-3'
Prl3a1 forward	Quantitative RT-PCR	5'-AAAGCAAGGAACTCCAGCAA-3'
Prl3a1 reverse	Quantitative RT-PCR	5'-TCAAGAGTAGCCAATGCACG-3'
Prl5a1 forward	Quantitative RT-PCR	5'-GTGGTGGGATTTTCCAGAGA-3'
Prl5a1 reverse	Quantitative RT-PCR	5'-TTCTGGCAACCTCCATCTTC-3'
Tpbpa forward	Quantitative RT-PCR	5'-TGTGCTGGTGTTCAGAGAAG-3'
Tpbpa reverse	Quantitative RT-PCR	5'-AGGTATATGGGAGAGTTTGTGG-3'
Prl8a8 forward	Quantitative RT-PCR	5'-ACTGAGGTCTATCCTACCTCAA-3'
Prl8a8 reverse	Quantitative RT-PCR	5'-TGTGGAGCGTCTCCAAAG-3'
Pcdh12 forward	Quantitative RT-PCR	5'-GTGGCTGAGCTTTACGGA-3'
Pcdh12 reverse	Quantitative RT-PCR	5'-AAACAGAGTGAGCTGCAGTT-3'
